# An *in vivo* detection system for transient and low‐abundant protein interactions and their kinetics in budding yeast

**DOI:** 10.1002/yea.3063

**Published:** 2015-02-10

**Authors:** Andrea Brezovich, Martina Schuschnig, Gustav Ammerer, Claudine Kraft

**Affiliations:** ^1^ Max F. Perutz LaboratoriesUniversity of ViennaViennaA‐1030Austria

**Keywords:** M‐Track, methylation, low‐abundant protein, histone 3, *Saccharomyces cerevisiae*

## Abstract

Methylation tracking (M‐Track) is a protein‐proximity assay in *Saccharomyces cerevisiae*, allowing the detection of transient protein–protein interactions in living cells. The bait protein is fused to a histone lysine methyl transferase and the prey protein to a methylation acceptor peptide derived from histone 3. Upon interaction, the histone 3 fragment is stably methylated on lysine 9 and can be detected by methylation‐specific antibodies. Since methylation marking is irreversible in budding yeast and only takes place in living cells, the occurrence of artifacts during cell lysate preparation is greatly reduced, leading to a more accurate representation of native interactions. So far, this method has been limited to highly abundant or overexpressed proteins. However, many proteins of interest are low‐abundant, and overexpression of proteins may interfere with their function, leading to an artificial situation. Here we report the generation of a toolbox including a novel cleavage‐enrichment system for the analysis of very low‐abundant proteins at their native expression levels. In addition, we developed a system for the parallel analysis of two prey proteins in a single cell, as well as an inducible methylation system. The inducible system allows precise control over the time during which the interaction is detected and can be used to determine interaction kinetics. Furthermore, we generated a set of constructs facilitating the cloning‐free genomic tagging of proteins at their endogenous locus by homologous recombination, and their expression from centromeric plasmids. GenBank submissions: pCK900; KM407502, pCK901; KM407503, pCK902; KM407504, pCK903; KM407505, pCK904; KM407506, pCK905; KM407507, pCK906; KM407508, pCK907; KM407509, pCK908; KM407510, pCK909; KM407511, pCK910; KM407512, pCK911; KM407513. © 2015 The Authors. *Yeast* published by John Wiley & Sons Ltd.

## Introduction

The analysis of transient protein–protein interactions, such as enzymes with their substrates and interactions of low‐abundant proteins, has proved to be difficult. Recently, a novel *in vivo* proximity assay, M‐Track, has been developed to overcome this restriction (Zuzuarregui *et al*., 2012). This method is based on fusing a histone lysine methyltransferase (HKMT) to a bait protein and its substrate, a peptide derived from histone 3 (H3), to a prey protein. Upon interaction, the prey is stably methylated on lysine 9 of H3 *in vivo*, which can be detected by methylation‐specific antibodies, even after the interacting proteins have separated. As a readout, western blotting of whole‐cell extracts was applied using monoclonal antibodies specific for methylation of lysine 9 on H3. This method has been successfully employed to detect transient protein–protein interactions, such as the binding of the phosphatase PP2A to its substrate Net1 (Zuzuarregui *et al*., 2012), which could not be detected previously. Similarly, the interaction of the Atg1 kinase with its substrate Atg9 could be visualized using M‐Track (Papinski *et al*., 2014). An additional advantage of M‐Track is that the readout mark, H3 methylation, is generated in living cells. This strongly reduces the occurrence of artifacts during cell lysate preparation. With the aim of expanding the usability of this proximity assay to very low‐abundant proteins, we established a prey‐enrichment system, independent of the prey protein's size. Here we describe this system, as well as a series of plasmids allowing the plasmid‐based and genomic tagging of proteins with H3 and HKMT. The centromeric plasmids can be used for amino‐terminal as well as carboxyl‐terminal tagging. The integration constructs allow the exchange of the GFP tag in the commercially available GFP library (Invitrogen) with H3 or HKMT by homologous recombination, without the necessity of cloning the protein of interest. We also developed a novel system allowing the tracking of two different prey proteins in parallel. In addition, an inducible methylation system was generated, in which FK506‐induced dimerization (Sio *et al.*, 2005) is used to induce methylation by tethering HKMT to the bait protein only upon FK506 addition.

## Materials and methods

### Plasmid construction

Plasmids are listed in Table 1 and a detailed cloning procedure is indicated in Tables S1 and S3 (see supporting information). A pFA6‐derived vector containing the GFP homology domain and a URA3‐resistance cassette, followed by a TEF terminator (van de Pasch *et al*., 2013), were used for replacement of the haemagglutinin (HA)–Cherry tag for four N‐terminal peptides of histone 3 (H3, ARTKQTARKSTGGKAPRKQL), followed by three HA epitope tags. Several rounds of mutagenesis resulted in the unique restriction sites *Pac*I, *Not*I, *Pme*I and *Sac*I, resulting in pLW48. Additional mutagenesis to generate a unique *Sbf*I site resulted in pCK901. The TEV protease recognition peptide (ENLYPQG) and two protein A sequences were introduced into the *Sbf*I site, resulting in pCK902. After several rounds of mutagenesis, the N‐terminal 2xHA region was removed and a unique *Sal*I was introduced after the remaining 3xHA region. URA3 was then excised via *Spe*I–*Sph*I and replaced with PCR‐amplified LEU2 from pRS315. The 4xH3–3xHA region was then excised with *Sbf*I–*Sal*I and replaced by 9xmyc–SUV, giving rise to pCK900.

**Table 1:** Plasmids used in this study

**Name**	**Characteristics**	**Promoter**	**ORF**	**Source**	**GenBank ID**
pCK900	Integrative mycHKMT tag, LEU2	–	–	This study	KM407502
pCK901	Integrative H3HA tag, URA3	–	–	This study	KM407503
pCK902	Integrative TEV–protA–H3HA tag, URA3	–	–	This study	KM407504
pCK903	C‐term mycHKMT tag, pRS415	Fsh2	Fsh2	This study	KM407505
pCK904	C‐term H3HA tag, pRS416	Atg18	Atg18	This study	KM407506
pCK905	C‐term TEV–protA–H3HA tag, pRS416	Atg18	Atg18	This study	KM407507
pCK906	C‐term TEV–protA–H3HA–HisFLAG tag, pRS413	Atg18	Atg18	This study	KM407508
pCK907	N‐term mycHKMT tag, pRS415Gal1	Gal1	MCS	This study	KM407509
pCK908	N‐term H3HA tag, pRS416Gal1–URA3*	Gal1	MCS	This study	KM407510
pCK909	N‐term H3–protA–TEV tag, pRS416Gal1–URA3*	Gal1	MCS	This study	KM407511
pCK910	C‐term CNB1, pRS415	Atg1	Atg1	This study	KM407512
pCK911	promADH–FKBP–mycHKMT, pRS413	ADH	FKBP	This study	KM407513
pRS315, pRS415	CEN LEU2	–	MCS	Sikorski and Hieter, 1989	
pRS316, pRS416	CEN URA3	–	MCS	Sikorski and Hieter, 1989	
pRS413	CEN HIS3	–	MCS	Sikorski and Hieter, 1989	
pRS413ADH	ADH promoter, CYC1 terminator, CEN HIS3	ADH	MCS	Mumberg *et al*., 1995	
pCK319	ATG1–GFP, pRS315	Atg1	Atg1	Kraft *et al*., 2012	
pCK371	ATG18–TAP, pRS315	Atg18	Atg18	Papinski *et al*., 2014	
pAB26	pRS416Gal1–URA3*, removed *Pst*I site in URA3	Gal1	MCS	This study	
pAB32	Atg1–TEV–protA–H3HA–HisFLAG, pRS413	Atg1	Atg1	This study	
pLW30.1	Atg1–HKMT, YCp111	Atg1	Atg1	This study	
pLW36	Pbs2–HKMT, YCp111	Pbs2	Pbs2	This study	
pLW38.1	Atg13–HKMT, YCp111	Atg13	Atg13	This study	
pLW42	Atg17–HKMT, YCp111	Atg17	Atg17	This study	
pLW48	Integrative H3HA tag, URA3, Sbf1 not unique	–	–	This study	
pLW52	Atg2–mycHKMT, pRS415	Atg2	Atg2	This study	
pMK80	RFP–FKBP:Nat			Gallego *et al*., 2013	

The sequences of pCK900‐911 were submitted to GenBank.

To generate centromeric plasmids, pCK371 (Papinski *et al*., 2014) was mutated to introduce a unique *Sbf*I site between the Atg18 ORF and the TAP tag. The 4xH3–3xHA tag was then subcloned via *Sbf*I–*Sal*I, replacing the TAP tag. The Atg18‐4xH3–3xHA insert was then further subcloned via *Not*I–*Sal*I into a pRS416 vector, in which the *Pst*I site in the URA3 region was mutated (URA3*), generating pCK904. pCK905 was generated by introducing a TEV cleavage site and two protein A sequences from pCK902 into the *Sbf*I site of pCK904. To generate pCK903, the ATG9‐encoding region of pLW49.1 (Papinski *et al*., 2014) was replaced via *Not*I–*Sbf*I by the ORF of FSH2 and 638 bases of its endogenous promoter, generating pCK903. A His–FLAG tag was then added into the *Sal*I site of pCK905 and subcloned to the HIS3‐containing vector pRS413, resulting in pCK906. The *Not*I–*Sbf*I region of pCK906 was then replaced by the ORF and promoter of ATG1, excised from pCK319 (Kraft *et al*., 2012), resulting in pAB32.

To generate vectors with N‐terminal methylation tags, 9xmyc–HKMT and 4xH3–3xHA were cloned into pRS415Gal1 or pRS416Gal1 vectors, respectively. 9xmyc–HKMT was PCR‐amplified with *Spe*I and *Sbf*I overhangs and introduced into the *Spe*I–*Pst*I sites of pRS415Gal1. Subsequent mutagenesis removed additional *Sac*I and *Pst*I sites, generating pCK907. The *Pst*I site in pRS416Gal1 was removed by mutagenesis, generating pAB26 (URA3*). The 4xH3–3xHA was synthesized and introduced into the *Spe*I–*Pst*I site of pAB26, generating pCK908. The 4xH3–protA–TEV tag was synthesized and introduced into the *Spe*I–*Pst*I site of pAB26. An additional *Sal*I site was removed, generating pCK909.

pCK910 was generated by replacing the GFP tag in pCK319 with PCR‐amplified CNB1 and introducing a CYC1 terminator. FKBP was PCR‐amplified and ligated into pRS413ADH via *Bam*HI–*Eco*RI. Subsequently, myc–HKMT was added via *Sbf*I–*Sal*I, generating pCK911.

All constructs were verified for expression and/or functionality in the respective deletion strain.

### Yeast strains and growth media

Yeast strains are listed in Table 2 and S2. Genomic tagging was achieved by homologous recombination of the M‐Track tags with the GFP tag in yeast strains available from Life Technologies (http://clones.lifetechnologies.com; Huh *et al.*, 2003). pCK900‐902, and pLW48 to generate yLW25 and yAB67, were used for the tag exchange. To allow homologous recombination, these plasmids were linearized with *Pac*I and *Pme*I (pCK900) or *Pac*I and *Sac*I or *Pme*I (pCK901/902) prior to transformation. This results in the expression of a protein of interest under its endogenous promoter at its genomic locus with a C‐terminal M‐Track tag.

**Table 2:** Yeast strains used in this study

**Name**	**Genotype**	**Background**	**Source**
BY4741	*his3Δ1 leu2Δ0 met15Δ0 ura3Δ0; Mat* **a**	BY474x	EUROSCARF
BY4743	*his3Δ1/his3Δ1 leu2Δ0/leu2Δ0 met15Δ0/MET15 ura3Δ0/ura3Δ0 lys2D0/LYS2; diploid*	BY474x	EUROSCARF
MKY2128	*his3Δ1 leu2Δ0 ura3Δ0 can1::Ste2pr‐Leu2 lyp1Δ tor1‐1 fpr1::klURA; Matα*	Y7039	Gallego *et al*., 2013
yAB2	*ATG17‐protAH3HA:URA*	BY474x	This study
yAB5	*ATG13‐protAH3HA:URA*	BY474x	This study
yAB7	*ATG17‐protAH3HA:URA atg13::KANMX6*	BY474x	This study
yAB66	*ATG1‐CNB1:NATMX6 fpr1::URA cnb1::KANMX6 ATG13‐protAH3HA:URA*	BY474x	This study
yAB67	*ATG17‐H3HA:URA atg13::KANMX6, MET15, Matα*	BY474x	This study
yAB68	*ATG17‐GFP:HISMX6*	BY474x	LifeTechnologies
yLW25	*ATG1‐H3HA:URA atg13::KANMX6*	BY474x	This study
yLW43	*ATG1‐protAH3HA:URA*	BY474x	This study
yTB12	*cnb1::KANMX6/CNB1; diploid*	BY474x	EUROSCARF
yTM26	*ATG1‐CNB1 cnb1::KANMX6 fpr1::URA, Matα*	BY474x	This study

All strains of *S.cerevisiae* S288C BY474x genetic background are derived from the diploid strain BY4743 and carry the following markers: *his3Δ1*, *leu2Δ0*, *met15Δ0*, *ura3Δ0*, *Mat* **a**, unless stated otherwise.

Yeast cells were grown in synthetic medium (SD; 0.17% yeast nitrogen base, 0.5% ammonium sulphate, 2% glucose, amino acids as required) or rich medium (YPD; 1% yeast extract, 2% peptone, 2% glucose). For autophagy induction, cells were grown to mid‐log phase and subsequently treated with 220 nm rapamycin for 1 h.

FKBP tethering to Cnb1 was induced by the addition of 300 nm FK506 (LC Laboratories) for 1 h to the growing cells, prior to harvesting.

### M‐Track assay

The M‐Track methylation assay was performed as described (Zuzuarregui *et al*., 2012). Protein extraction with TCA was carried out as described previously (Kijanska *et al.*, 2010). To prepare an extract under non‐denaturing conditions, cells equivalent to OD_600_ × ml = 150 were harvested by centrifugation. Cell extracts were prepared by glass bead lysis. Prey proteins (methylated or unmethylated) were enriched by immunoprecipitation on IgG‐bound magnetic beads. The 32 kDa H3 tag and the 37 kDa H3–flag tag were separated from the prey protein by TEV cleavage: protein bound to beads was incubated with TEV protease lysis buffer containing 0.5 mm DTT and TEV protease for 1 h at 16°C. The supernatant was removed and bead‐bound protA–H3 tags were then analysed for trimethylation by western blotting. Note that both tags migrate on western blots at a slightly larger size. Anti‐trimethylation antibodies are sold by different companies, such as Novus Biologicals and Merck Millipore: rabbit anti‐trimethylation antibodies are suitable for extract blots; however they should be avoided for enrichment studies, due to their strong cross‐reactivity with protein A present in the M‐Track enrichment constructs. Although several mouse antibodies show only little binding to protein A and were successfully used for the enrichment studies in this study, some mouse IgG subclasses, such as IgG_2_, bind protein A efficiently and are therefore likely not suited for these enrichment approaches.

## Results and discussion

**Figure 1:**
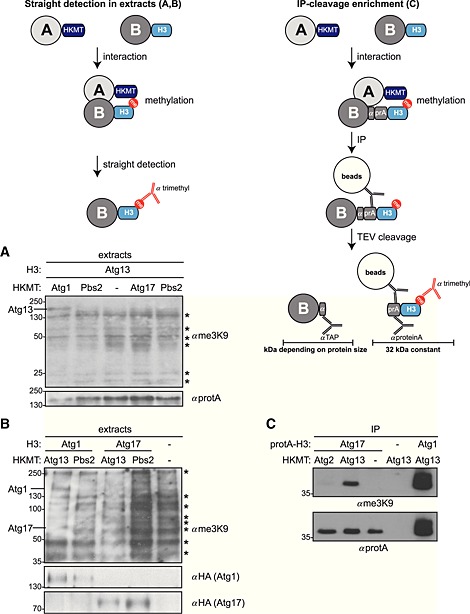
M‐Track signal enrichment system. Illustrations explaining the M‐Track signal enrichment systems used: (A) Atg13–protA–H3 yeast cells expressing plasmid‐based HKMT‐tagged Atg1, Atg17, Pbs2 or an empty plasmid (pRS315) were grown to mid‐log phase and TCA precipitated. Extracts were analysed by anti‐me3K9 and anti‐protA western blotting. (B) Wild‐type, Atg17–H3 or Atg1–H3 yeast strains containing HKMT‐tagged Atg13, Pbs2 or an empty plasmid (pRS315) were grown to mid‐log phase and treated with rapamycin for 1 h, which induces autophagy and possibly increases the interaction of proteins analysed. Extracts were prepared and analysed as described in (A). (C) Wild‐type, Atg17–protA–H3 or Atg1–protA–H3 yeast strains containing HKMT‐tagged Atg2, Atg13 or an empty plasmid (pRS315) were grown to mid‐log phase and treated with rapamycin for 1 h. Extracts were prepared and protA–H3 tagged proteins were isolated on IgG magnetic beads, followed by TEV cleavage. The protA–H3 tags bound on the beads were analysed by western blotting.

The protein proximity assay M‐Track has been described for the study of short‐lived protein–protein interactions in living cells (Zuzuarregui *et al*., 2012). Whereas M‐Track was successfully applied for the analysis of short‐lived protein interactions, such as kinase/phosphatase–substrate bindings that had failed in conventional approaches (Zuzuarregui *et al*., 2012; Kraft *et al*., 2012; Papinski *et al*., 2014), the analysis of interactions with very low‐abundant proteins under physiological conditions has not been assessed. To establish M‐Track for the latter, we used the known interactions of Atg13 with Atg1 and Atg17 to set up the system. Atg1, Atg13 and Atg17 are proteins essential in the autophagy pathway and have been previously described to interact (Kabeya *et al.*, 2005). To compare proteins of different abundance in M‐Track, we analysed the Atg1–Atg13 and Atg17–Atg13 interaction pairs. Atg1 is a low‐abundant protein of about 1000 molecules/cell and Atg17 shows even lower expression levels of 300 molecules/cell (www.yeastgenome.org). Both proteins have been shown to interact with Atg13. To assess the interaction of Atg13 with Atg1 and Atg17 *in vivo*, we fused Atg13 to H3 and Atg1 and Atg17 to HKMT and analysed their interactions by anti‐trimethylation (me3K9) immunoblotting of cell extracts. Pbs2 served as a negative control, as this protein has never been observed to bind to Atg13. As previously reported, Atg1 showed a specific interaction with Atg13 (Kraft *et al*., 2012) (Figure 1A). Similarly, inverting the methylation tags resulted in Atg13–HKMT interacting with Atg1–H3 in cell extracts (Figure 1B). However, in both cases no specific band could be detected for the interaction between Atg13 and Atg17 (70 kDa) and several unspecific bands appeared (*) that complicated interpretation, suggesting that this proximity assay is not suitable for very low‐abundant proteins.

### Cleavage‐enrichment system for visualizing low‐abundant proteins in M‐Track

To adapt M‐Track for the analysis of very low‐abundant proteins, we aimed to generate a prey enrichment system. As several non‐specific bands appear on anti‐me3K9 immunoblots on whole‐cell extracts (Figure 1A and B, asterisks), the size of the prey protein can influence detection efficiency. In addition, transfer efficiency of proteins during western blotting varies with protein size. We therefore aimed to generate a system which functions independent of prey protein size, allowing direct comparison of prey proteins of different molecular weight. For efficient enrichment, we added a protein A (protA) tag to the H3 prey peptide, allowing rapid isolation of the prey protein on magnetic IgG beads (Figure 1C). In addition, a TEV cleavage site was placed between the protein of interest and the protA–H3 tag, to allow the separation of the protein (of varying size) from the methylated tag (of constant size) for analysis (Figure 1C). The size of the cleaved methylation tag was chosen to be approximately 32 kDa, a size range free of non‐specific bands on anti‐me3K9 western blots of yeast extracts (Figure 1A). A short peptide recognized by the commercial anti‐TAP antibody (indicated by *α*; Pierce CAB1001) was added upstream of the TEV cleavage site, which allows the detection of the cleaved free prey protein.

We tested this novel tag for analysing the Atg17–Atg13 interaction, which we were unable to detect by straight immunoblotting (Figure 1A and B). Atg17 was fused to the cleavable protA–H3 tag and Atg13 was fused to HKMT. As a negative control, Atg2, which is not expected to interact with Atg13, or non‐tagged proteins were used. The Atg1–Atg13 interaction served as a positive control. Cell extracts were prepared and Atg17 was isolated on IgG magnetic beads, followed by TEV cleavage. Methylation on the cleaved protA–H3 tag bound to the beads was analysed by anti‐me3K9 immunoblotting, and the overall amount of the cleaved tag was assessed by anti‐protA detection. As shown in Figure 1C, the interaction of Atg17 with Atg13 could readily be visualized in this enrichment system, and an even stronger methylation signal was detected for the Atg1–Atg13 control interaction. These findings suggest that the cleavage‐enrichment system allows the analysis of very low‐abundant proteins by M‐Track. The size‐independent readout of the M‐Track signal furthermore allows a better comparison of different prey proteins of varying size. Note that the cleaved tag of calculated 32 kDa runs at a slightly larger size on western blots.

**Figure 2:**
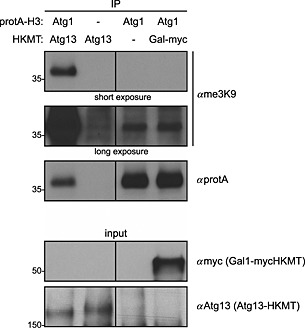
Methylation‐independent cross‐reaction of mouse IgGs with protein A. Wild‐type or Atg1–protA–H3 yeast cells containing Atg13–HKMT, myc–HKMT expressed from a Gal1 promoter (pCK907) or an empty plasmid (pRS315) as indicated were grown to mid–log phase and treated with rapamycin for 1 h. Protein isolation, TEV cleavage and western blotting was performed as in Figure 1C. Note that an unspecific background signal is visible in lane 3 and 4 of the me3K9 western blot due to the high expression of the protA tag and its unspecific binding to the antibody used for western blotting. Input extracts used for immunoprecipitation are also shown. The panels separated by a black line come from the same blot with the same exposure.

ProtA interacts strongly with rabbit IgGs, whereas several subtypes of mouse IgGs tend to show only a low affinity for protA. Therefore the use of anti‐me3K9 antibodies raised in rabbits should be avoided when using the protA–H3 tags. To test for spurious methylation and to detect the methylation‐independent background signal, an empty plasmid containing no HKMT and HKMT fused to 9xmyc under a strong Gal1 promoter were used (Figure 2). A weak band is also visible after longer exposure in the absence of any HKMT, suggesting that this band is methylation‐independent and originates from the cross‐reaction of mouse IgGs with protein A (Figure 2, lanes 3 and 4). This band showed no clear increase when HKMT was overexpressed (lane 4), suggesting that, if at all, only little spurious methylation occurs.

### Analysis of two prey proteins in parallel

To allow the parallel analysis of two prey proteins in the same cell, we generated an additional cleavable H3 methylation acceptor tag by adding a 6xHis–4xFLAG epitope. This shifts the molecular weight from 32 to 37 kDa, enabling the separation of the two H3 tags by SDS–PAGE. Parallel expression of two prey proteins carrying the H3 tags of different size allows their parallel investigation with a single HKMT tagged bait.

**Figure 3:**
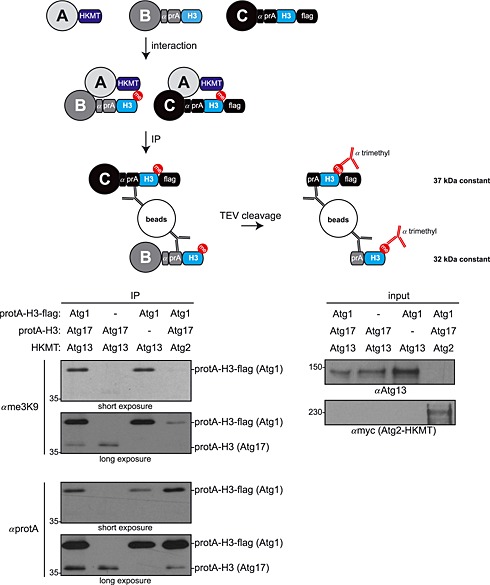
Monitoring two prey proteins in parallel. Atg17–protA–H3 yeast cells containing Atg1–protA–H3–FLAG, Atg13–HKMT, Atg2–myc–HKMT or an empty plasmid (pRS315) as indicated were grown to mid–log phase and treated with rapamycin for 1 h. Protein isolation, TEV cleavage and western blotting was performed as in Figure 1C. Note that an unspecific background signal is visible in lane 4 of the me3K9 western blot due to the high expression of the protA tag and its unspecific binding to the antibody used for western blotting. Input extracts used for immunoprecipitation are also shown.

Atg13 is known to interact with both Atg1 and Atg17 (Kabeya *et al*., 2005). We monitored both interactions in parallel by co‐expressing Atg13–HKMT, Atg17–protA–H3 and Atg1–protA–H3–FLAG in the same cell. As shown in Figure 3, both Atg1 and Atg17 were specifically methylated on their H3 tags that were well separated by SDS–PAGE, due to their difference in size. These findings show that, in combination, the novel cleavable H3 tags are suitable for the detection of two prey proteins in the same cell.

### Inducible methylation system

**Figure 4:**
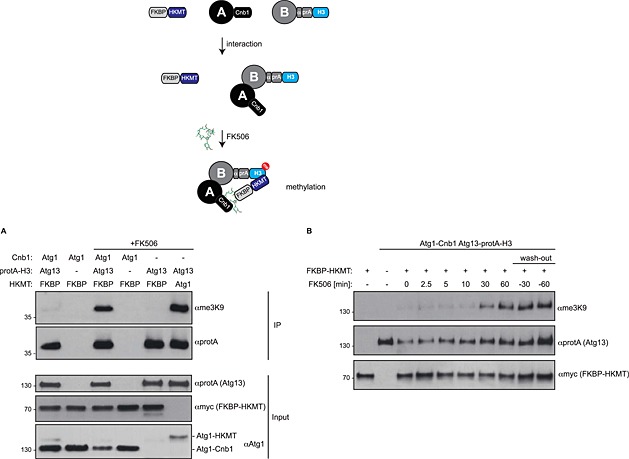
Inducible M–Track system. (A) Atg13–protA–H3 Atg1–Cnb1 or Atg13–protA–H3 yeast cells containing FKBP–HKMT or Atg1–HKMT on a centromeric plasmid were grown to mid‐log phase and treated with FK506 for 1 h. FK506 tethers HKMT to Cnb1. Protein isolation, TEV cleavage and western blotting was performed as in Figure 1C. Input extracts used for immunoprecipitation are also shown. (B) Atg13–protA–H3 Atg1–Cnb1 or wild‐type yeast cells containing FKBP–HKMT or an empty plasmid (pRS315) were grown to mid‐log phase, treated with FK506 for the indicated times, TCA precipitated and analysed by western blotting. FK506 was washed out by transferring the cells to fresh medium for the indicated times.

If the bait and the prey proteins interact constitutively, H3 methylation will continuously accumulate and finally reach saturation. This may impede or even preclude analysis of differences in binding strength. To allow the analysis of the order of binding events as well as the strength of interaction, we constructed an inducible M‐Track system. We used the binding of Cnb1 to FKBP upon FK506 addition (Sio *et al*., 2005) to induce recruitment of HKMT to the bait protein. To test our system, we used it to monitor the interaction between Atg1 and Atg13. Atg13 was fused to the protA–H3 methylation acceptor tag, Atg1 was tagged with Cnb1 and FKBP–HKMT was expressed from a centromeric plasmid. As shown in Figure 4A, no methylation of Atg13–protA–H3 could be detected without FK506. After FK506 addition for 1 h, a specific me3K9 signal on the H3 tag could be visualized, which almost reached the intensity of the constitutive interaction (compare lanes 3 and 6 in Figure 4A). A time course showed that after 30 min a strong signal had already appeared, which only increased slightly when incubation was prolonged for another 30 min. The methylation mark did not decrease within 60 min of removing FK506 from the medium (Figure 4B). This could result from either the non‐reversible binding of FK506 to FKBP and Cnb1 or the stability of the methylation mark, which will only be lost when diluted by sufficient cell divisions after the wash‐out of FK506. Taken together, in contrast to the constitutive M‐Track methods described above, this M‐Track system can indeed be ‘turned on’ by inducing the recruitment of HKMT to the bait protein, and allows the measurement of interactions at the minute scale. This opens new possibilities for the applicability of this method and offers the analysis also of kinetic events.

### Genomic integration system

**Figure 5:**
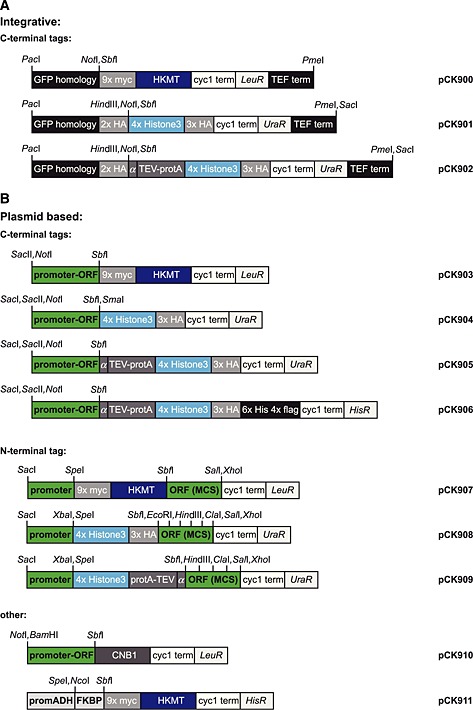
M‐Track tagging toolbox. (A) Constructs for genomic tagging of yeast strains with HKMT, H3 or protA–H3. Unique restriction sites are indicated. (B) Centromeric plasmid toolbox for C‐ and N‐terminal M‐Track tagging. Unique restriction sites are indicated.

**Figure 6:**
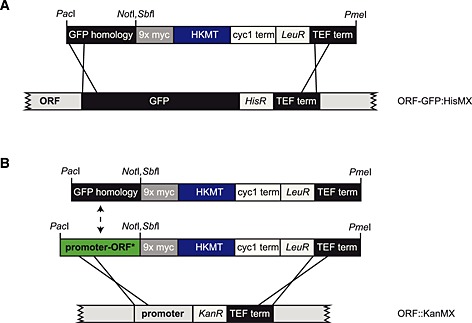
Cloning‐free integration of HKMT and H3 tags by homologous recombination. (A) A GFP homology domain and the TEF terminator allow the recombination of the methylation tag with the GFP tag in the GFP library strains available from Life Technologies. (B) Alternatively, a gene of interest (or a mutant form) including its endogenous promoter can be cloned into the *Pac*I and *Not*I or *Sbf*I sites, allowing recombination via the promoter and the TEF terminator on the plasmid and also present in the KanMX ORF deletion library from EUROSCARF.

To facilitate tagging and allow simple genomic integration, we constructed plasmids allowing homologous recombination with the GFP library distributed by Life Technologies (http://lifetechnologies.com; Huh *et al*., 2003) (Figure 5A). Homologous recombination is achieved via a 33 base pair N‐terminal region on the targeting plasmid homologous to the N‐terminus of the GFP tag in the library and the TEF terminator present in both the GFP library strains and the targeting plasmid (Figure 6A). The plasmids are linearized by *Pac*I and/or *Pme*I to allow recombination to occur. Successful recombinants are selected for the gain of uracil or leucine auxotrophy and loss of the ability to grow on medium lacking histidine. Further verification of recombination can be monitored by immunoblotting the recombinants for the myc tag present in the HKMT constructs and the protA or the HA tag fused to the H3 domain.

To allow the integration of mutant proteins with HKMT or H3 tags, the GFP‐homology domain can be replaced by the promoter and mutant ORF (ORF*) of a protein of interest. Transformation into the respective yeast deletion library strain (EUROSCARF, http://web.uni‐frankfurt.de/fb15/mikro/euroscarf) allows homologous recombination between the promoter and the TEF terminator, replacing the KanMX cassette with the mutant protein tagged with HKMT or H3 (Figure 6B).

### M‐Track tagging toolbox

In addition to the integration constructs, we generated a set of centromeric plasmids to further facilitate protein analysis using M‐Track. Both N‐ and C‐terminal fusion constructs of HKMT and H3 were made (Figure 5B, Table 1).

In summary, we describe here new M‐Track based techniques, allowing *in vivo* detection of transient interactions between low‐abundant proteins and analysis of their kinetics in budding yeast. A collection of plasmids for genomic and plasmid based expression of yeast proteins with different H3 and HKMT tags for M‐Track analysis were constructed. The integration constructs are compatible with homologous recombination into commercially available yeast libraries, providing a useful tool for further yeast proteome studies. This technique might also be applicable in other organisms in the future.

## Supporting information




**Table S1**. Detailed cloning procedure.
**Table S2**. Yeast strain generation.
**Table S3**. Primers used in this study.

Supporting info item
