# Supplementary material for: An in vivo detection system for transient and low‐abundant protein interactions and their kinetics in budding yeast
Source: Yeast. 2015 Feb 10;32(3):355–65. doi: 10.1002/yea.3063 (PMC4949564; doi:10.1002/yea.3063)
Supplement: Supplementary file 1 — Supporting info item [file YEA-32-355-s001.docx]

**Supplementary Table S1.** Detailed cloning procedure

| **Name** | **Cloning** |  |  |
| --- | --- | --- | --- |
| pCK670 | The *Sbf*I site in pLW48 was removed by mutagenesis with oLW36 and oLW37. The backbone was PCR amplified and blunt ligated with oMS1 and oMS3, resulting in the removal of the N-terminal 2xHA and *Hind*III region. The *Sal*I site was then removed with oMS4 and oMS5, and a new *Sal*I was introduced with oMS6 and oMS7. |  |  |
| pCK900 | The URA3 marker in pCK670 was replaced with the LEU2 marker from pRS315 by PCR amplification with oCK390 and oCK391 and exchanged via *Spe*I*-Sph*I. The H3HA region was then replaced by the 9xmycHKMT region of pLW49.1 [Papinski et al. 2014] using *Sbf*I*-Sal*I. |  |  |
| pCK901 | pLW48 was mutagenized with oLW36 and oLW37. |  |  |
| pCK902 | The TEV-protA sequence was PCR amplified with oCK329 and oCK330 from pCK320 [Kraft et al. 2012] and ligated via *Pst*I into pCK901. |  |  |
| pCK903 | FSH2 was PCR amplified from genomic DNA with 636 bases of its endogenous promoter using oCK483 and oCK484 and ligated via *Not*I*-Sbf*I into pLW49.1 [Papinski et al. 2014]. |  |  |
| pCK904 | The 5' 2xHA region in pCK901 was removed by amplifying the backbone and blunt ligation with primers oMS1 and oMS3. The *Sal*I site after the CYC1 terminator was then removed with primers oMS4 and oMS5. A new *Sal*I site was introduced after the H3 tag by amplifying the backbone and blunt ligation using primers oMS6 and oMS7. The 4xH3-3xHA insert was then subcloned via *Sbf*I*-Sal*I into pCK371 [Papinski et al. 2014], which had been modified with oCK327 and oCK328 to change the *Pst*I site to *Sbf*I. The *Not*I*-Sal*I insert was then subcloned to pRS416 [Sikorski and Hieter 1989], in which the *Pst*I site in the URA gene had been removed with primers oLW36 and oLW37. |  |  |
| pCK905 | TEV-protA sequence of pCK902 was excised with *Pst*I and ligated into the *Sbf*I site of pCK904. |  |  |
| pCK906 | A 6xHis4xFlag sequence was synthesized (Eurofins) and subcloned via *Xho*I*-Sal*I into the *Sal*I site of pCK905. The *Sac*I*-Sal*I insert was then subcloned to pRS413ADH. |  |  |
| pCK907 | 9xmyc-HKMT was PCR amplified from pLW49.1 [Papinski et al. 2014] with oligos oCK395 and oCK396 with *Spe*I and *Sbf*I overhangs and introduced into the SpeI*-Pst*I sites of pRS415Gal1 [Mumberg, Müller, and Funk 1994]. Subsequent mutagenesis with oligos oAB12, oAB13, oAB16, oAB17, oAB18 and oAB19 removed additional *Sac*I and *Pst*I sites. |  |  |
| pCK908 | The 4xH3-3xHA sequence was synthesized (Eurofins) and introduced into the *Spe*I*-Pst*I site of pAB26. |  |  |
| pCK909 | The 4xH3-protA-TEV sequence was synthesized (Eurofins) and introduced into the *Spe*I*-Pst*I site of pAB26. An additional *Sal*I site was removed with oligos oAB23 and oAB24. |  |  |
| pCK910 | CNB1 was PCR amplified from vector 406 (pFA6a CNB1:NatMX) kindly provided by Robbie Loewith and used in [Shimada et al. 2013] using primers oTM2 and oTM3 and ligated via *Pst*I*-Sal*I into pCK319. The CYC1 terminator was added via *Xho*I*-Kpn*I excised from pRS413ADH. |  |  |
| pCK911 | FKBP was PCR amplified from pMK80 [Gallego, Specht, Brach, Kumar, Gavin, and Kaksonen 2013] using primers oAB14 and oAB15 and subcloned via *Bam*HI*-Eco*RI into pRS413ADH. mycHKMT from pLW49.1 [Papinski et al. 2014] was then added via *Sbf*I*-Sal*I*.* |  |  |
| pAB26 | The *Pst*I site in the URA gene of pRS416Gal1 [Mumberg, Müller, and Funk 1994] was modified with primers oLW36 and oLW37. |  |  |
| pAB32 | The *Not*I*-Sbf*I region of pCK906 was replaced by the *Not*I*-Pst*I region of pCK319 [Kraft et al. 2012]. |  |  |
| pLW30.1 | The *Xba*I*-Sph*I backbone of PC7 [Zuzuarregui et al. 2012] containing HKMT was annealed with preannealed oCK310 and oCK311. One *PstI* site was mutated with oLW5 and oLW6. This vector was cut with *Not*I and *Pst*I, and Atg1 with its endogenous promoter was subcloned from pCK320 [Kraft et al. 2012] via these sites. |  |  |
| pLW38.1 | The *Xba*I*-Sph*I backbone of PC7 [Zuzuarregui et al. 2012] containing HKMT was annealed with preannealed oCK310 and oCK311. One *Pst*I site was mutated with oLW5 and oLW6. The vector was cut with *Not*I*-Pst*I and ATG13 was PCR amplified from genomic DNA with 698 bases of its endogenous promoter using oCK157 and oCK158, and ligated via these sites. |  |  |
| pLW42 | ATG17 was amplified with oLW22 and oLW23 from the Molecular Barcoded Yeast (MoBY) ORF Library ATG17 clone (GE Healthcare). |  |  |
| pLW48 | A pFA6 derived vector containing the GFP homology domain and a URA3 resistance cassette followed by a TEF terminator [van de Pasch et al. 2013] was used as backbone. A PCR with primers oLW31 and oLW32 of the H3HA tag was ligated into the *Hind*III and *Kpn*I sites of this backbone. The H3HA plasmid PI225 [Zuzuarregui et al. 2012] was mutagenized to remove duplicate restriction sites using oLW1, oLW2, oLW3, oLW4, oLW15 and oLW16 prior to the PCR. |  |  |
| pLW52 | ATG2 was PCR amplified with 777 bases of its endogenous promoter with oCK151 and oCK152 from genomic DNA and cloned via *Not*I*-Sbf*I into pLW49.1 [Papinski et al. 2014]. |  |  |

**Supplementary Table S2.** Yeast strain generation

| **Name** | **Genotype** | **Generation** |  |
| --- | --- | --- | --- |
| yAB2 | *ATG17-protAH3HA:URA* | The GFP tag of ATG17-GFP (LifeTechnologies) was replaced by homologous recombination using transformation of *Pac*I*-Sac*I linearized pCK902. |  |
| yAB5 | *ATG13-protAH3HA:URA* | The GFP tag of ATG13-GFP (LifeTechnologies) was replaced by homologous recombination using transformation of *Pac*I*-Sac*I linearized pCK902. |  |
| yAB7 | *ATG17-protAH3HA:URA atg13::KANMX6* | yAB2 was crossed to atg13::KANMX6 (Euroscarf). |  |
| yAB66 | *ATG1-CNB1:NATMX6 fpr1::URA cnb1::KANMX6 ATG13-protAH3HA:URA* | yAB5 was crossed with yTM26. |  |
| yAB67 | *ATG17-H3HA:URA atg13::KANMX6, MET15, Mat alpha* | The GFP tag of ATG17-GFP (LifeTechnologies) was replaced by homologous recombination using transformation of *Pac*I*-Sac*I linearized pLW48, and crossed to atg13::KANMX6 (Euroscarf). |  |
| yLW25 | *ATG1-H3HA:URA atg13::KANMX6* | ATG1-GFP (LifeTechnologies) was crossed to atg13::KANMX6 (Euroscarf). The GFP tag was replaced by homologous recombination using transformation of *Pac*I*-Sac*I linearized pLW48. |  |
| yLW43 | *ATG1-protAH3HA:URA* | The GFP tag of ATG1-GFP (LifeTechnologies) was replaced by homologous recombination using transformation of *Pac*I*-Sac*I linearized pCK902. |  |
| yTM26 | *ATG1-CNB1:NATMX6 cnb1::KANMX6 fpr1::URA, Mat alpha* | FPR1 was replaced by klURA cassette from MKY2128 using primers oTB200 and oTB201. The fpr1::URA strain was crossed to cnb1::KANMX6 (Euroscarf). ATG1 was C-terminally tagged with CNB1 using primers oCK1 and oTB134 and plasmid 406 kindly provided by Robbie Loewith and used in [Shimada et al. 2013]. |  |

**Supplementary Table S3.** Primers used in this study

| **Pirmer** | **Sequence** |  |
| --- | --- | --- |
| oAB12 | CTTGAACGGATCCACTCTACAGGAGTCTAGGGGATC |  |
| oAB13 | GATCCCCTAGACTCCTGTAGAGTGGATCCGTTCAAG |  |
| oAB14 | AAAAGGATCCATGGGAGTGCAGGTGGAAACCA |  |
| oAB15 | AAAAGAATTCGGCCTGCAGGGATTCCAGTTTTAGAAGCTCCACATCGAAG |  |
| oAB16 | CTGGGAGCAGGAGCTAAATGCCAAGCGCAG |  |
| oAB17 | CTGCGCTTGGCATTTAGCTCCTGCTCCCAG |  |
| oAB18 | GGGCAGGCGAGGAGCTGACCTTTGATTACAAC |  |
| oAB19 | GTTGTAATCAAAGGTCAGCTCCTCGCCTGCCC |  |
| oAB23 | GCACAAGCACCTAAAGTCGATAACAAGTTCAATAAGGAACAG |  |
| oAB24 | CTGTTCCTTATTGAACTTGTTATCGACTTTAGGTGCTTGTGC |  |
| oCK1 | CAGGTTGAAAATATTGAGGCAGAAGATGAACCACCAAAATCGGATCCCCGGGTTAATTAA |  |
| oCK151 | AAGCGGCCGCTTAATCACTACTGCTAGTAAG |  |
| oCK152 | AACCTGCAGGGAATCAGTCCGATTGGACTT |  |
| oCK157 | AAGCGGCCGCTGGGCAGGATTGACAGAGC |  |
| oCK158 | AACCTGCAGGCCTTCTTTAGAAAGGTTCATA |  |
| oCK310 | CGAGCTCCACCGCGGTGGCGGCCGCTCTAGATTAATTAACCTGCAGGTCGACCTCGAGA |  |
| oCK311 | CTAGACTCGAGGTCGACCTGCAGGTTAATTAATCTAGAGCGGCCGCCACCGCGGTGGAGCTCGCATG |  |
| oCK327 | CCATCTTGATGGACCTGCAGGGACGGATCCCCGG |  |
| oCK328 | CCGGGGATCCGTCCCTGCAGGTCCATCAAGATGG |  |
| oCK329 | AACCTGCAGGAGAAAATCTCATCCTCCGGGG |  |
| oCK330 | AACTGCAGCTGATGATTCGCGTCTACTTTC |  |
| oCK390 | AAAACTAGTCCTCGAGGAGAACTTCTAGT |  |
| oCK391 | AAAGCATGCTTAAGCAAGGATTTTCTTAACTTCTT |  |
| oCK395 | AAAACTAGTATG CAGGGTTCTGCTGCTAGTG |  |
| oCK396 | AAACCTGCAGG GAAGAGGTATTTGCGGCAGG |  |
| oCK483 | AAAACCTGCAGGGTCAACTGCTGGAGCCAT |  |
| oCK484 | AAAAGCGGCCGCGTGAGAGAGAAAATGCGAATGG |  |
| oLW1 | GGCCCAGATCTGCGGACGCATCTTTTACC |  |
| oLW2 | GGTAAAAGATGCGTCCGCAGATCTGGGCC |  |
| oLW3 | GCTGCTCAGTGCGGACGCTAGGATCC |  |
| oLW4 | GGATCCTAGCGTCCGCACTGAGCAGC |  |
| oLW5 | GTTGTGACCCCAACCTACAGGTGTACAACGTC |  |
| oLW6 | GACGTTGTACACCTGTAGGTTGGGGTCACAAC |  |
| oLW15 | CCTCGAGTCTAGGGGATCCCCGGGG |  |
| oLW16 | CCCCGGGGATCCCCTAGACTCGAGG |  |
| oLW22 | TTGCGGCCGCTTCGCCTAAAACCTCC |  |
| oLW23 | TTCTGCAGAGGATTCTTCACGTTGTAATTT |  |
| oLW31 | AAAAGCTTGCGGCCGCCCTGCAGGTCGACCTCG |  |
| oLW32 | TTGGTACCCTAGCGTCCGCACTGAGC |  |
| oLW36 | CCCAACTGCACAGAACAAAAACATGCAGGAAACG |  |
| oLW37 | CGTTTCCTGCATGTTTTTGTTCTGTGCAGTTGGG |  |
| oMS1 | GCGGCCGCCCTGCAGGACGAC |  |
| oMS3 | CTCGAGTCCAGTGAAAAGTTC |  |
| oMS4 | GGTACTAGTCAGGACGACGCGGCATCAG |  |
| oMS5 | CTGATGCCGCGTCGTCCTGACTAGTACC |  |
| oMS6 | GACTAGGGTACCCAGCTTTTGTG |  |
| oMS7 | GACGCACTGAGCAGCGTAATC |  |
| oTB134 | AGCAGGTCATTTGTACTTAATAAGAAAACCATATTATGCATCACTTAATCGATGAATTCGAGCTCG |  |
| oTB200 | ACCCTGAATACAAGGAAGC |  |
| oTB201 | GTGAGGCAGAAGGTAAAGG |  |
| oTM2 | AAACCTGCAGGGTATGGGTGCTGCTCCTTCCAAA |  |
| oTM3 | AAAGTCGACTTACACATCGTATTGCAATGTCAG |  |

**Supplementary references**

Gallego O, Specht T, Brach T, Kumar A, Gavin A-C, Kaksonen M. 2013. Detection and Characterization of Protein Interactions In Vivo by a Simple Live-Cell Imaging Method Keen J. (ed). *PLoS ONE*, **8**: e62195.

Kraft C et al. 2012. Binding of the Atg1/ULK1 kinase to the ubiquitin-like protein Atg8 regulates autophagy. *EMBO J*, **31**: 3691–3703.

Mumberg D, Müller R, Funk M. 1994. Regulatable promoters of Saccharomyces cerevisiae: comparison of transcriptional activity and their use for heterologous expression. *Nucleic Acids Res.*, **22**: 5767–5768.

Papinski D et al. 2014. Early Steps in Autophagy Depend on Direct Phosphorylation of Atg9 by the Atg1 Kinase. *Mol Cell*, **53**: 471–483. http://eutils.ncbi.nlm.nih.gov/entrez/eutils/elink.fcgi?dbfrom=pubmed&id=24440502&retmode=ref&cmd=prlinks.

Shimada K et al. 2013. TORC2 signaling pathway guarantees genome stability in the face of DNA strand breaks. *Mol Cell*, **51**: 829–839.

Sikorski RS, Hieter P. 1989. A system of shuttle vectors and yeast host strains designed for efficient manipulation of DNA in Saccharomyces cerevisiae. *Genetics*, **122**: 19–27.

van de Pasch LAL et al. 2013. Centromere binding and a conserved role in chromosome stability for SUMO-dependent ubiquitin ligases. *PLoS ONE*, **8**: e65628.

Zuzuarregui A et al. 2012. M-Track: detecting short-lived protein-protein interactions in vivo. *Nat Meth*, **9**: 594–596.
